# *Vibrio* Flagellar Synthesis

**DOI:** 10.3389/fcimb.2019.00131

**Published:** 2019-05-01

**Authors:** Mylea A. Echazarreta, Karl E. Klose

**Affiliations:** Department of Biology, South Texas Center for Emerging Infectious Diseases, The University of Texas at San Antonio, San Antonio, TX, United States

**Keywords:** *Vibrio*, flagella, motility, chemotaxis, biofilm, virulence

## Abstract

*Vibrio* spp. are highly motile Gram-negative bacteria, ubiquitously found in aquatic environments. Some *Vibrio*s are responsible for disease and morbidity of marine invertebrates and humans, while others are studied for their symbiotic interactions. *Vibrio* spp. are motile due to synthesis of flagella that rotate and propel the bacteria. Many *Vibrio* spp. synthesize monotrichous polar flagella (e.g., *V. cholerae, V. alginolyticus*); however, some synthesize peritrichous or lophotrichous flagella. Flagellar-mediated motility is intimately connected to biological and cellular processes such as chemotaxis, biofilm formation, colonization, and virulence of *Vibrio* spp. This review focuses on the polar flagellum and its regulation in regard to *Vibrio* virulence and environmental persistence.

## Introduction

*Vibrio* spp. are highly motile Gram-negative bacteria that are ubiquitous in aquatic environments. Some *Vibrio*s are responsible for human illnesses such as cholera (*V. cholerae*), vibriosis (*V. parahaemolyticus*), and wound infections (*V. vulnificus*), while others are studied for their symbiotic interactions (*V. fischeri*). Motility of *Vibrio* spp. is achieved through rotation of flagella, which are literally “whip”-like appendages that facilitate swimming. Flagellar-mediated motility is intimately connected to processes such as chemotaxis, biofilm formation, colonization, and virulence of *Vibrio* spp. (McCarter, 2004; Butler and Camilli, 2005; Yildiz and Visick, 2009; Yoon et al., 2012; Teschler et al., 2015; Wang et al., 2017). Thus, motility plays a significant role in the lifestyle of *Vibrios*, both in the aquatic environment and during host colonization. Many *Vibrio* spp. are monotrichous with a single, sheathed polar flagellum (e.g., *V. cholerae* and *V. alginolyticus*); however, some *Vibrios* can also be peritrichous or lophotrichous. This review focuses on the sheathed polar flagellum, which requires over 50 proteins to synthesize the unique appendage that plays such an integral role in *Vibrio* biology (Zhu et al., 2013).

## Flagellar Structure

**Figure 1:**
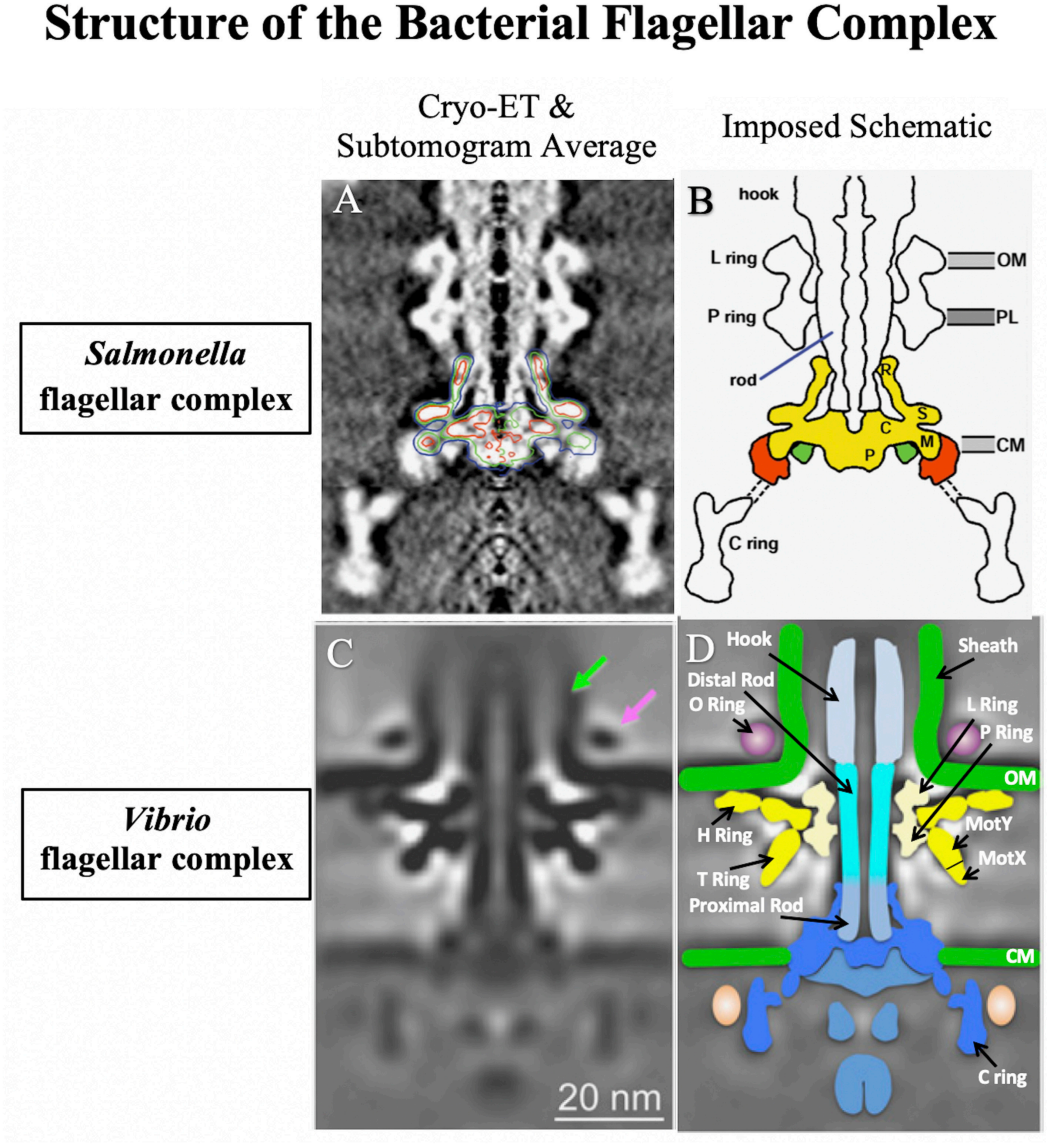
A comparison of the well-studied *Salmonella enterica* serovar Typhimurium flagellar complex (Suzuki et al., 2004) to that of *Vibrio alginolyticus* (Zhu et al., 2017). The flagellar complex was imaged using cryoelectron tomography (Cryo-ET) combined with genetic subtomogram analysis. Final reconstructed images were generated via superimposed density maps. **(A)** Electron microscopy rendering of the *S*. Typhimurium flagellar complex with the basal body outlined. **(B)** Schematic of the *S*. Typhimurium flagellar complex. **(C)** Electron microscopy rendering of the sheathed *V. alginolyticus* flagellar complex. *Vibrio* specific attributes are depicted by arrows: the sheath (green arrow) and the O ring (purple arrow). **(D)** Schematic of the sheathed *V. alginolyticus* flagellar complex. Images reproduced and modified with permission.

The bacterial flagellum is typically described as being composed of three major structural components: the basal body, the hook and the filament. Each major structural component is assembled in a hierarchical manner starting at the inner cytoplasmic membrane, proceeding to the outer membrane, and ultimately outside the cell. The best-studied bacterial flagella are the peritrichous flagella of *Salmonella enterica*, so it is instructive to contrast *S. enterica* flagella with the *Vibrio* polar flagella (Figure 1).

In *S. enterica*, the basal body is anchored within the inner membrane by the MS Ring (FliF) and associated export apparatus within the cytosol, referred to as the C ring (FliG, FliM, and FliN), with a helical rod (FlgB, FlgC, FlgF, and FlgG) that spans the periplasmic space (Figures 1A,B). The rod also has rings associated with the two layers it passes through: a P ring (FlgI) associated with the peptidoglycan layer, and an L ring (FlgH) associated with the outer membrane. Motor proteins (MotA and MotB) that allow the flagellum to spin by utilizing the proton motive force are associated with the MS ring and are also referred to as the stator. Outside the outer membrane, the hook connects the basal body to the flagellar filament. The hook is composed of FlgE protein, and is a flexible coupler between the spinning basal body and the flagellar filament. Finally, the filament is a long hollow tube composed of a single flagellin subunit that adopts a helical shape that, when the motor is spinning counterclockwise, bundle together with the other peritrichous flagella to propel the cell forward. The filament has a capping protein (FliD) at the distal end to facilitate correct folding of the flagellins as they exit the secretion channel and to prevent them from being secreted into the extracellular space. The *S. enterica* flagellar structure has been described in detail in a number of excellent reviews (Aizawa, 1996; Chilcott and Hughes, 2000; Morimoto and Minamino, 2014; Minamino and Imada, 2015).

The *Vibrio* polar flagellum shares many similarities with *S. enterica* flagella (Figures 1C,D). However, some of the notable differences include the number of flagella, polar placement, energetics, additional components of the basal body, multiple flagellin subunits and the presence of a sheath surrounding the filament. Much of what is known about the *Vibrio* polar flagellar structure has been studied in elegant detail in *V. alginolyticus*, along with some studies in *V. cholerae* and a few other *Vibrio* spp. We will discuss these differences here:

### Flagellar Number and Placement

Two proteins control flagellar number and placement in *Vibrio* spp.: FlhF and FlhG. These proteins are also found in other polarly flagellated bacteria. Inactivation of *flhG* results in *Vibrio*s with multiple polar flagella, whereas inactivation of *flhF* results in either no flagella or occasional cells with a misplaced flagellum at a location other than the pole (Correa et al., 2005). Inactivation of both *flhF* and *flhG* results in some cells having multiple peritrichous flagella, demonstrating that these proteins control flagellar number (FlhG) and placement (FlhF). FlhF is a GTP-binding protein that localizes to the old cell pole and recruits FliF, the earliest structural component of the flagellum (Green et al., 2009). The GTP-bound form of FlhF forms dimers, whereas the GDP-bound form is a monomer (Kondo et al., 2017). FlhF mutations that prevent GTP-binding inhibit flagellar synthesis whereas mutations that prevent GTP hydrolysis do not (Green et al., 2009; Kondo et al., 2017), leading to the hypothesis that GTP-binding allows dimerization and polar localization.

FlhG is an ATPase that shares homology with the cell division regulator MinD (Correa et al., 2005). FlhG acts antagonistically with FlhF: overproduction of FlhG or depletion of FlhF decreases flagellar number, whereas depletion of FlhG or overproduction of FlhF increases flagellar number. FlhG in the cytoplasm appears to sequester FlhF and prevent it from polar localization (Kusumoto et al., 2008). It has been proposed that the ATP-bound form of FlhG localizes to the cell pole and interacts directly with polarly-localized FlhF, decreasing its affinity for the pole; upon ATP hydrolysis the FlhG-FlhF complexes diffuse into the cytoplasm (Ono et al., 2015). However, FlhG does not intrinsically localize to the pole, but rather interacts with the polar landmark protein HubP (Takekawa et al., 2016). An additional protein, SflA, also interacts with HubP and suppresses the formation of lateral flagella in cells lacking FlhF and FlhG (Inaba et al., 2017). SflA is a transmembrane protein with a DnaJ domain, but its mechanism of action in inhibiting lateral flagellar synthesis is not yet clear (Kitaoka et al., 2013).

### Energetics

Motor torque that drives flagellar rotation is generated by interaction between the rotor in the basal body and the non-rotating stator complex that surrounds the rotor. Rotation of the flagella of *S. enterica* and many other bacteria is driven by a H^+^ gradient that flows through the stator channel in the flagellar motor composed of MotA and MotB. In contrast, the *Vibrio* polar flagellum rotates due to a Na^+^-driven motor. The Na^+^-driven *Vibrio* flagellum can rotate at speeds more than five times faster than the H^+^-driven flagella of *E. coli* (Magariyama et al., 1994; Chen and Berg, 2000).

The Na^+^-driven motor contains the additional components MotX and MotY, which form the T ring found in the *Vibrio* flagellar basal body (Figure 1D). The T ring is composed of MotY extending from the P ring complexed with MotX (Zhu et al., 2017). The T ring extends to contact the stator proteins MotA and MotB (also called PomA and PomB). The T ring stabilizes the MotA/MotB stator around the rotor; in the absence of MotX or MotY the MotA and MotB proteins do not localize to the cell pole (Terashima et al., 2006). Mixing Na^+^-specific MotA/MotB with H^+^-specific MotA/MotB has demonstrated that Na^+^ specificity lies in the MotB component (Asai et al., 1999).

### The H Ring

The *Vibrio* basal body contains an additional ring, the H ring, which extends from the L ring and is associated with the outer membrane (Figure 1D). The H ring is composed of FlgT and FlgO: FlgT is in direct contact with the LP and T rings, and FlgO is at the distal end of FlgT in contact with the OM (Zhu et al., 2018). An additional protein FlgP is a lipoprotein associated with the outer membrane (Morris et al., 2008) that is required for motility. This protein is also likely associated with the H ring, since the H ring is missing in the *V. fischeri* basal body lacking FlgP (Beeby et al., 2016). It has been hypothesized that the H ring structure is part of the stator and is required to generate the high torque that drives the *Vibrio* motor (Beeby et al., 2016). Interestingly, *V. cholerae* lacking FlgT or FlgP appear non-motile in semi-solid agar (Cameron et al., 2008; Morris et al., 2008; Martinez et al., 2010), whereas *V. cholerae flgO* mutants only exhibit reduced motility in this medium (Martinez et al., 2009). The *flgO, flgP*, and *flgT* mutants still synthesize flagella, but the filaments appear fragile in *flgO* and *flgP* mutants, and easily detached in *flgT* mutants (Morris et al., 2008; Martinez et al., 2009, 2010). Lipidation of FlgP is required for outer membrane localization, but not for flagellar function, suggesting that its suspected role in stator assembly oddly does not require membrane localization.

### Multiple Flagellin Subunits

The filament of the *S. enterica* flagellum is made of a single flagellin protein. In contrast, *Vibrios* synthesize flagellar filaments composed of multiple flagellin subunits, typically 5-6 different flagellin proteins. *V. cholerae* has a dominant flagellin, FlaA, that is required and sufficient for filament formation, although the other four flagellins (FlaBCDE) can also be found within the filament (Klose and Mekalanos, 1998a; Yoon and Mekalanos, 2008). The gene corresponding to *flaA* in other *Vibrios* (*V. vulnificus, V. parahaemolyticus, V. fischeri, and V. anguillarum)* is not essential for motility (McCarter, 1995; Milton et al., 1996; Millikan and Ruby, 2004; Kim et al., 2014), although all *flaA* mutants show some level of decreased motility.

*V. cholerae flaA* mutants show decreased intestinal colonization (Martinez et al., 2009), *V. anguillarum flaA* mutants are defective for virulence in fish and a *V. fischeri flaA* mutant is defective for symbiotic colonization of the squid (Milton et al., 1996; Millikan and Ruby, 2004). *V. cholerae* can synthesize a monoflagellin filament with only FlaA. None of the other four flagellins are capable of this due in part to the presence of a lysine residue at position 145 within a putative beta sheet that is predicted to be involved in flagellin-flagellin interactions (Echazarreta et al., 2018). This suggests that FlaA may play an important role not only for filament structure but also in facilitating the other flagellins to be incorporated into the filament. It is still not clear why the other flagellins are incorporated into the filament if they are not needed for flagellar synthesis and motility, but they may impart more subtle changes to swimming behavior. All the flagellins can stimulate TLR5-dependent inflammatory responses (Xicohtencatl-Cortes et al., 2006; Harrison et al., 2008; Yoon and Mekalanos, 2008; Rui et al., 2010).

### Flagellar Sheath

The *Vibrio* flagellum has a sheath that surrounds the filament, which is an extension of the outer membrane that is composed of LPS (Fuerst and Perry, 1988) (Figure 1C, green arrow). Secretion of the anti-sigma factor FlgM through the filament has been detected, suggesting that there may be a sheath opening at the flagellar tip (Correa et al., 2004; Liu et al., 2008). Biogenesis of the flagellar sheath remains mysterious, but some unsheathed flagella have been visualized in *Vibrios* engineered to express multiple flagella by a *flhG* mutation (Zhu et al., 2017). Moreover, an outer membrane ring (O ring) can be seen by cryoelectron tomography at the base of the hook on the outside of the sheath (Zhu et al., 2017) that the authors speculate may be involved in sheath formation (Figure 1C, magenta arrow). Interestingly, *Vibrios* lacking FlgT synthesize some flagella that fail to penetrate the outer membrane, forming periplasmic flagella and suggesting that the H ring plays a role in sheath formation (Zhu et al., 2018). The function of the sheath also remains a mystery; the sheath was proposed to shield immunogenic flagellins from the immune system (Yoon and Mekalanos, 2008; Rui et al., 2010). However, in *V. fischeri*, rotation of the sheathed flagella causes blebs of LPS that are important for triggering host immune response in the squid (Brennan et al., 2014).

## Flagellar Transcription Hierarchy

**Figure 2:**
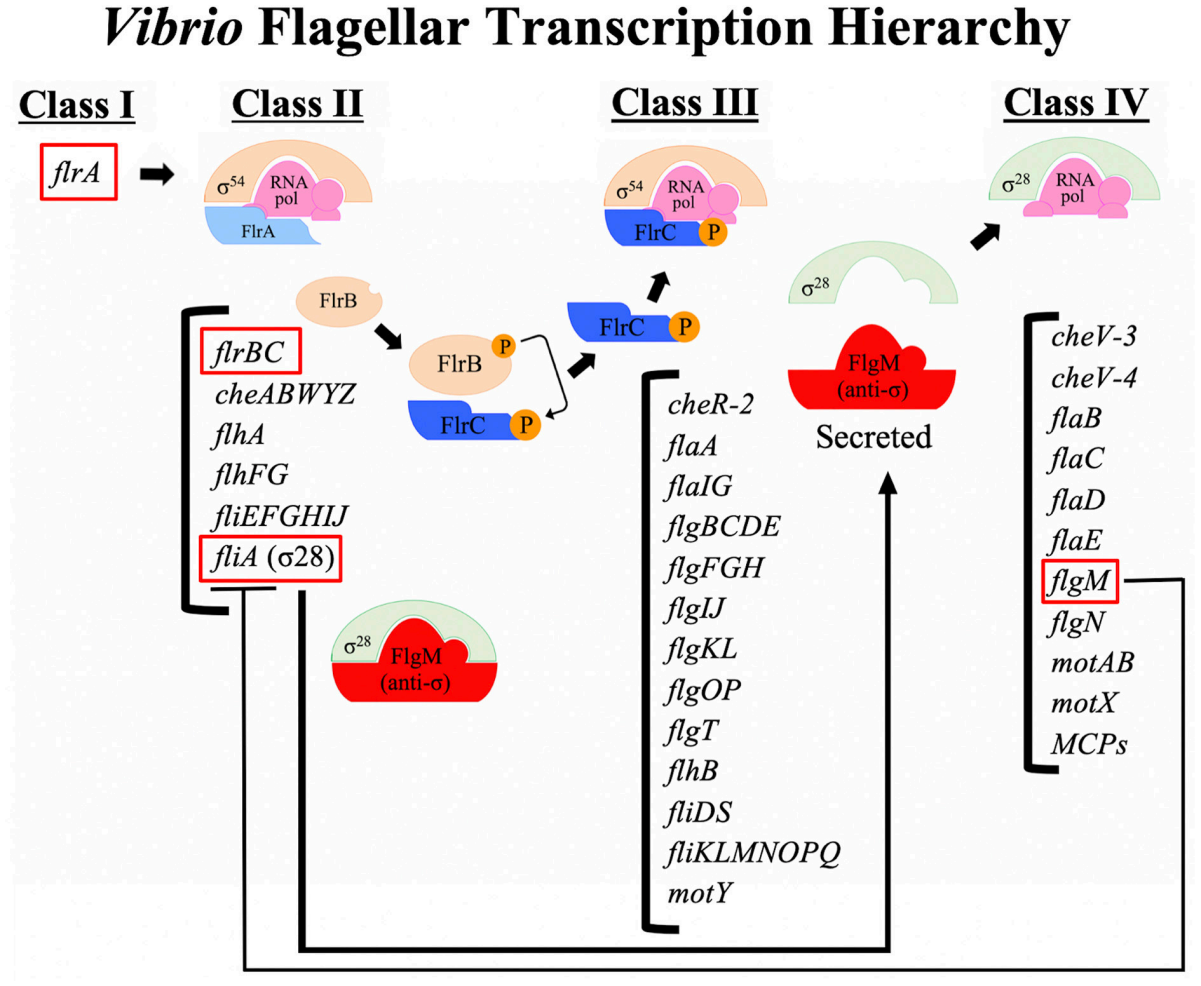
A schematic of the flagellar transcription hierarchy of *Vibrio cholerae*. *V. cholerae* flagellar genes are expressed within a four-tiered transcriptional hierarchy. The sole Class I gene of the *Vibrio* hierarchy is the σ^54^-dependent transcriptional activator, *flrA*, which is the master regulator of the flagellar hierarchy. The FlrA- and σ^54^-dependent Class II genes encode components of the MS ring, C ring, ATPase and export apparatus associated with the cytoplasmic membrane, components of the chemotaxis machinery, along with proteins that determine the location and number of flagella (discussed in text). The FlrC- and σ^54^-dependent Class III genes encode additional components of the C ring, inner membrane export apparatus, the rod, rod cap, PLH and T rings, torque generator, hook components, filament cap, the FlaA flagellin, the flagellin chaperone, a chemotaxis protein, and proteins of unknown function. The σ^28^-dependent Class IV genes encode additional flagellins, motor components, chemotaxis proteins, and methyl-accepting chemoreceptors (MCPs).

The flagellum is assembled in a stepwise fashion starting with the export apparatus in the cytoplasmic membrane, which then secretes components in the correct order to form the basal body and hook, and ending with secretion of large amounts of flagellin protein to form the filament. Flagellar gene transcription is regulated to allow transcription to also occur in a stepwise fashion, resulting in a flagellar transcription hierarchy where components of the flagellum that are needed early in assembly (e.g., export components) are transcribed earlier than components needed later in assembly (e.g., flagellin). Moreover, late gene transcription is dependent not only on successful transcription of the early components but also the successful assembly of these components. The three-tiered flagellar transcription hierarchy of *S. enterica* has been elegantly elucidated and described (Kutsukake et al., 1990; Dasgupta et al., 2003; Chevance and Hughes, 2008). The flagellar transcription hierarchy of *Vibrio*s has been most studied in *V. cholerae* and is distinct from that of *S. enterica* (Figure 2).

*V. cholerae* flagellar genes are expressed within a four-tiered transcriptional hierarchy (Klose and Mekalanos, 1998b). The *Vibrio* flagellar hierarchy involves transcription by RNA polymerase containing the alternate sigma factor σ^54^, which requires enhancer-binding transcriptional activators in order to initiate transcription (Buck et al., 2000). The *Vibrio* flagellar hierarchy also involves transcription by RNA polymerase containing the alternate sigma factor σ^28^, similar to the *S. enterica* hierarchy. The four-tiered *Vibrio* hierarchy is most similar to that of other monotrichous bacteria (e.g., *P. aeruginosa* and *A. hydrophila*) (Dasgupta et al., 2003; Wilhelms et al., 2011).

The sole Class I gene of the *Vibrio* hierarchy is the σ^54^-dependent transcriptional activator, *flrA* (Klose and Mekalanos, 1998b; Prouty et al., 2001; Syed et al., 2009). FlrA is the master regulator of the flagellar hierarchy because without it no flagellar genes are expressed. The histone-like nucleoid structuring protein (H-NS) positively regulates transcription of the *flrA* gene, while simultaneously negatively regulating *ctx* and *tcp* gene expression (Ghosh et al., 2006). FlhG, which controls flagellar number (see above), negatively regulates *flrA* transcription (Correa et al., 2005). FlrA-dependent activation of Class II flagellar genes is modulated by c-di-GMP (Srivastava et al., 2013). C-di-GMP binds directly to FlrA and disrupts FlrA binding to Class II promoters, which in turn prevents activation of Class II gene transcription (Srivastava et al., 2013). High c-di-GMP levels are known to drive *V. cholerae* into the sessile biofilm lifestyle (Jones et al., 2015, discussed below).

The FlrA- and σ^54^-dependent Class II genes include components of the MS ring (FliF), C ring (FliG), ATPase (FliHIJ) and export apparatus (FlhA) associated with the cytoplasmic membrane, along with FlhF and FlhG which determine the location and number of flagella (discussed above). Components of the chemotaxis machinery (CheABWYZ) are also expressed as Class II genes. Importantly, the regulatory proteins that control Class III (FlrBC) and Class IV (FliA) are expressed as Class II genes. Interestingly, additional components that are required for secretion across the cytoplasmic membrane are expressed as Class III genes (described below); the *fliKLMNOPQRflhB* operon is transcribed from a Class III promoter (Syed et al., 2009), suggesting that Class II transcription alone does not result in a secretion competent apparatus. It is unclear what signal allows transcription to shift from Class II to Class III transcription, but it is likely not secretion of an inhibitor.

FlrC is a σ^54^-dependent transcriptional activator that activates the transcription of Class III flagellar genes (Klose and Mekalanos, 1998b; Prouty et al., 2001; Syed et al., 2009). FlrC is also a response regulator and member of a two-component regulatory system along with the histidine kinase FlrB (Correa et al., 2000). FlrB autophosphorylates itself and then transfers the phosphate to a conserved aspartate residue (D54) within the amino terminus of FlrC (Correa et al., 2000; Prouty et al., 2001). FlrC-P then activates σ^54^-dependent transcription of Class III flagellar genes (Correa et al., 2000). The FlrC binding sites within the promoter regions, although typically located downstream of the start site of transcription, act as true enhancers, i.e., they can be moved upstream and still stimulate transcription (Correa and Klose, 2005). The central portion of FlrC is the σ^54^-activation domain (Klose and Mekalanos, 1998b), which contains the ATPase activity required to stimulate transcription by the σ^54^-holoenzyme (Weiss et al., 1991). This domain must be oligomerized to form the active ATPase conformation, and this usually happens upon phosphorylation of the N-terminal domain and binding of ATP, as with *S. enterica* NtrC (Porter et al., 1993). The structure of the FlrC σ^54^-activation domain revealed that it forms a heptamer in both nucleotide-free and -bound states without any ATP-dependent remodeling (Dey et al., 2015), unlike other σ^54^-dependent activators. Presumably, phosphorylation of FlrC by FlrB only occurs when some structural intermediate has been assembled, but this has not been identified yet. FlrB is a soluble protein, unlike many histidine kinases which are membrane-bound, so the signal may also be soluble, or perhaps FlrB interacts transiently with the nascent flagellar structure.

The FlrC- and σ^54^-dependent Class III genes include additional components of the C ring (FliMN), inner membrane export apparatus (FliOPQR and FlhB), proximal rod (FlgBCF), distal rod (FlgG), rod cap (FlgJ), P ring (FlgI), L ring (FlgH), H ring (FlgOT and FlgP, discussed above), T ring (MotY), torque generator (FliL), hook (FlgE), hook cap (FlgD), hook length control (FliK), hook-filament junction (FlgKL), filament cap (FliD), the flagellin chaperone (FliS), a chemotaxis protein (CheR-2), and proteins of unknown function (FlaGI and VC1384). Interestingly the FlaA flagellin is also transcribed as a Class III gene, despite the fact that this is a structural component of the filament (Prouty et al., 2001). Normally flagellin subunits are transcribed at high levels only after the synthesis and assembly of the hook-basal body complex, due to σ^28^-dependent transcription (Class IV genes in *Vibrio*s, described below). The FlaA flagellin is capable of forming a monoflagellin filament in the absence of the other flagellins, but it can do this even when expressed as a Class IV gene (Echazarreta et al., 2018), so it is unclear why it is normally expressed as a Class III gene. Additionally, a *flaA* mutant strain is not repressed for transcription of Class IV genes (Klose and Mekalanos, 1998a), indicating that the switch from Class III to Class IV expression (i.e., FlgM secretion, described below) occurs prior to the expression and assembly of FlaA into the filament. Dysregulation of Class III gene expression by a hyperactive (FlrB-independent) FlrC leads to decreased intestinal colonization (Correa et al., 2000) by an unknown mechanism.

The Class IV genes are transcribed by RNA polymerase with the alternate σ^28^ sigma factor (FliA) (Prouty et al., 2001). σ^28^-dependent late flagellar gene transcription is common to most bacterial flagellar transcription hierarchies including in *S. enterica* (Chilcott and Hughes, 2000). σ^28^ typically regulates the expression of flagellin genes, which are the structural subunits of the filament and are needed in large amounts, but only when the basal body-hook complex has been assembled. This is accomplished by the anti-sigma factor FlgM binding to σ^28^ and preventing σ^28^-dependent transcription until completion of the basal body-hook, at which time FlgM is secreted through the flagellum, allowing σ^28^ to associate with RNAP and activate transcription (Chadsey et al., 1998). This same mechanism for controlling σ^28^-dependent transcription is present in *V. cholerae* (Correa et al., 2004); FlgM binds to σ^28^ and prevents Class IV gene transcription until it is secreted through the flagellum. FlgM can be found in the supernatant of secretion competent flagellated bacteria, indicating that despite being sheathed, the *Vibrio* flagellum has some type of opening to allow secretion of FlgM into the supernatant.

The Class IV genes include the four flagellin proteins FlaBCDE, the stator (motor) proteins (MotA/PomA and MotB/PomB) as well as the motor protein MotX (T ring), chemotaxis proteins (CheV-3 and CheV-4) and at least 7 methyl-accepting chemoreceptors (MCPs) (Syed et al., 2009). The anti-σ^28^ factor FlgM also demonstrates the expression pattern of a Class IV gene (Prouty et al., 2001; Syed et al., 2009), but there must be sufficient expression of FlgM during Class II gene expression to repress the Class II FliA until the Class III-Class IV switch (Correa et al., 2004). The four Class IV flagellin proteins are not essential for filament formation (Klose and Mekalanos, 1998a) but are found within the flagellar filament, suggesting they may contribute more subtle attributes to filament formation (Yoon and Mekalanos, 2008).

## Motility and Virulence

Motility has been linked to the virulence of a number of different *Vibrios* (Milton et al., 1996; Yang et al., 2014). A non-motile *V. vulnificus* strain lacking all six flagellin genes is defective for adhesion to and invasion of HeLa cells and exhibits a 100-fold increase in LD_50_ in mice (Kim et al., 2014). Additionally, non-motile *V. vulnificus* mutants are defective in a murine wound model (Yamazaki et al., 2019). A non-flagellated mutant of the coral pathogen *V. coralliilyticus* is unable to chemotax toward, adhere to, or infect the coral *Pocillopora damicornis* (Meron et al., 2009). Non-motile mutants of *V. anguillarum*, a fish pathogen, are defective at invasion of fish cells *in vitro* and infection of rainbow trout *in vivo* (McGee et al., 1996; Ormonde et al., 2000). Flagellar synthesis and motility are under quorum sensing control in *V. harveyi*, a major pathogen of a number of different aquatic organisms, and inhibition of motility leads to decreased virulence in brine shrimp larvae (Yang and Defoirdt, 2015). *V. harveyi* sensing the catecholamine stress hormones norepinephrine (NE) and dopamine (Dopa) has been linked to increased growth, upregulation of flagellar genes, increased swimming motility, siderophore and exopolysaccharide production, and biofilm formation (Yang et al., 2014). *V. harveyi* pre-exposed to NE or Dopa are more virulent, suggesting that stress hormones present in shrimp hemolymph increases motility, virulence and transmission of *V. harveyi* (Yang et al., 2014). Motility is also important for the symbiotic colonization of the Hawaiian bobtail squid (*Euprymna scolopes*) by *V. fischeri* (Millikan and Ruby, 2002).

Non-motile mutants of *V. cholerae* are impaired for colonization of the infant mouse small intestine, but the level of impairment in this model seems to vary depending on the specific strain and the motility defect (Gardel and Mekalanos, 1996; Lee et al., 2001). Non-motile *V. cholerae* mutants were also shown to be defective for adherence to intestinal epithelia and stimulated lower levels of luminal fluid accumulation in rabbits (Freter et al., 1981; Richardson, 1991; Silva et al., 2006). Importantly, motility is critical for virulence of *V. cholerae* in humans. The induction of protective anti-*V. cholerae* O antigen (OAg) antibodies are the basis of immunity to cholera infection (Wang et al., 2017; Islam et al., 2018). Anti-OAg antibodies specifically prevent motility of *V. cholerae* by binding to the sheathed flagellum, and thus the basis of immunity to *V. cholerae* infection is the prevention of flagellar-based motility (Wang et al., 2017). Additionally, human volunteers orally vaccinated with live attenuated strains of *V. cholerae* experienced disease symptoms referred to as “reactogenicity” (Coster et al., 1995; Kenner et al., 1995), and this has been demonstrated to be caused by the expression of the flagellins, which stimulate an inflammatory response by TLR5 signaling (Rui et al., 2010).

There is evidence for inverse regulation of motility and virulence genes in *V. cholerae* (Gardel and Mekalanos, 1996; Syed et al., 2009; Syed and Klose, 2011; Rugel and Klose, 2012). The *V. cholerae* ToxR regulon responds to environmental stimuli to regulate ToxT expression, which then activates transcription of the virulence-associated cholera toxin (*ctx*), toxin co-regulated pilus (*tcp*), and accessory colonization factor (*acf* ) genes (Childers and Klose, 2007). Transcription profiling demonstrated upregulation of a number of these virulence genes, such as *ctx* and *tcp*, in non-motile *V. cholerae* strains (Syed and Klose, 2011). Additionally, a hemolysin (TLH; Fiore et al., 1997) and the HapA zinc-dependent metalloprotease are negatively regulated by flagellar synthesis/motility (Silva et al., 2006), while a hemagglutinin (FRH; Syed et al., 2009) is positively regulated. The FRH is positively regulated by the diguanylate cyclase (DGC) CdgD, which in turn is positively regulated by the Class IV flagellar regulator σ^28^ (FliA). FrhA is a surface adhesin that binds to epithelial cells and chitin, in addition to erythrocytes, and enhances intestinal colonization (Syed et al., 2009), thus linking the fucose-sensitive HA activity that was described previously (Gardel and Mekalanos, 1996) to a flagellar-regulated adhesin.

These opposing patterns of gene expression have led to a model where *V. cholerae* requires flagellar gene expression and chemotaxis (reviewed below) to arrive at the site of colonization within the intestine, but after arrival flagellar gene expression is downregulated while virulence genes like *ctx* and *tcp* are upregulated to allow colonization and disease. The normal site of *V. cholerae* colonization is within the intestinal crypts within the small intestine, and the crypts are covered by a thick layer of mucus. Liu et al. (2008) showed that *V. cholerae* lose their flagella when swimming through mucus, and this leads to repression of *hapR* transcription by σ^28^, which in turn would lead to enhanced *ctx* and *tcp* expression upon arrival at the epithelial cell surface. The *V. cholerae* flagellum functions as a Type III secretion apparatus for the flagellar structural proteins, but it was recently described to also secrete a cytotoxin. The motility-associated killing factor MakA is secreted through the flagellar channel and causes toxicity in both *Caenorabdis elegans* and zebrafish (Dongre et al., 2018) and thus potentially also within the intestine.

## Chemotaxis and Virulence

Bacterial chemotaxis is the movement toward a chemoattractant or away from a chemorepellent, and is therefore intimately connected to motility. Chemotaxis signal transduction has been reviewed in depth in bacteria, such as *E. coli* and *S. enterica* serovar Typhimurium, and is similar to that in *Vibrios* (Boin et al., 2004). Briefly, methyl-accepting chemoreceptor proteins (MCPs) and some MCP-like proteins (MLPs) in the cytoplasmic membrane bind chemoattractants, which induces a conformational change that stimulates signal transduction (Falke and Hazelbauer, 2001). The chemosignal is transmitted through CheW, a cytoplasmic linker protein, to protein kinase CheA (Gegner et al., 1992; Schuster et al., 1993). CheA autophosphorylates itself, and CheA-P transfers its phosphate to the response regulator CheY under inhibitory conditions. CheY-P then binds to the C-ring of the flagellar motor, transducing the environmental signal to alter flagellar rotation from counterclockwise (CCW) to clockwise (CW) rotation, and this causes the bacteria to reorient their swimming behavior. In peritrichous bacteria this results in changing from smooth swimming to tumbling behavior.

In *Vibrios*, chemotaxis is mediated by CCW forward propulsion and CW reversal of locomotion, subsequently reorienting the cell. However, unlike the run and tumble chemotactic behavior seen in peritrichously flagellated bacteria, *Vibrios* utilize a 3-step flick chemotactic search pattern characterized by a cycle of smooth-swimming forward, then a reversal of direction, followed by a 90° rotational flick (Winn et al., 2013). The flick allows *Vibrios* to undergo a consistent change in direction that allows for more rapid nutrient location and utilization in comparison to a run and tumble style of locomotion (Winn et al., 2013). In the presence of bound chemoattractant (e.g., amino acids, etc.), the flick cycle is modulated to increase the frequency of smooth-swimming forward to the source of the attractant (Ushijima and Hase, 2018). Chemotaxis can be important for *Vibrios* (e.g., *V. coralliilyticus* and *V. fischeri*) to locate a favorable environment and mediate successful colonization of a host (Graf et al., 1994; DeLoney-Marino et al., 2003; Ushijima and Hase, 2018). Non-chemotactic mutants of *V. anguillarum* are attenuated for infection, non-chemotactic *V. fischeri* are impaired for colonization of the Hawaiian bobtail squid (*Euprymna scolopes*), and *V. coralliilyticus* chemotaxes toward coral mucus (Millikan and Ruby, 2002; Butler and Camilli, 2005; Ushijima and Hase, 2018).

Unlike *E. coli* and *S*. Typhimurium*, V. cholerae* (and other *Vibrios*) has multiple chemotactic paralogues (Camilli and Mekalanos, 1995; Boin et al., 2004; Szurmant and Ordal, 2004). The *V. cholerae* genome contains three chemotaxis operons; however, the genes required for chemotactic signaling *in vitro* (e.g., *cheA-2* and *cheY-3*) are located within operon 2 (Butler and Camilli, 2005). CheA-1 (operon 1) and cheA-3 (operon 3) are dispensable, as are the other four *cheY* paralogs (*cheY*-1,−2,−4, and−5). Because operons 1 and 3 appear to not be required for control of chemotactic influenced flagellar motility, it is suspected that these genes may play a role in flagellum-independent chemotactic locomotion, such as surface migration (Brown and Hase, 2001; Butler and Camilli, 2005).

Chemotaxis plays an important role in *V. cholerae* gastrointestinal colonization. CW-biased non-chemotactic mutants are characterized by zig-zag motility with little net movement due to very frequent changes in direction of locomotion, whereas CCW-biased non-chemotactic mutants are associated with an increased frequency of smooth-swimming and longer straight runs. Interestingly, CW-biased *V. cholerae* bacteria are defective at intestinal colonization in the infant mouse, whereas the CCW-biased bacteria are hyperinfectious, significantly outcompeting wildtype strains within the intestine (Butler and Camilli, 2004). The CCW-biased *V. cholerae* exhibited an increase in infectivity, which was correlated to an expanded capacity for intestinal colonization that spanned the length of the small intestine. Excreted stool *V. cholerae* have also been shown to be non-chemotactic and hyperinfectious in the infant mouse model, similar to CCW-biased mutants (Butler et al., 2006). Stool *V. cholerae* maintain their transient competitive advantage after several hours of incubation in freshwater, suggesting that this hyperinfectivity caused by repression of chemotaxis is a driver of *V. cholerae* epidemic spread (Merrell et al., 2002; Butler and Camilli, 2004; Butler et al., 2006).

## Motility and Biofilm Formation

**Figure 3:**
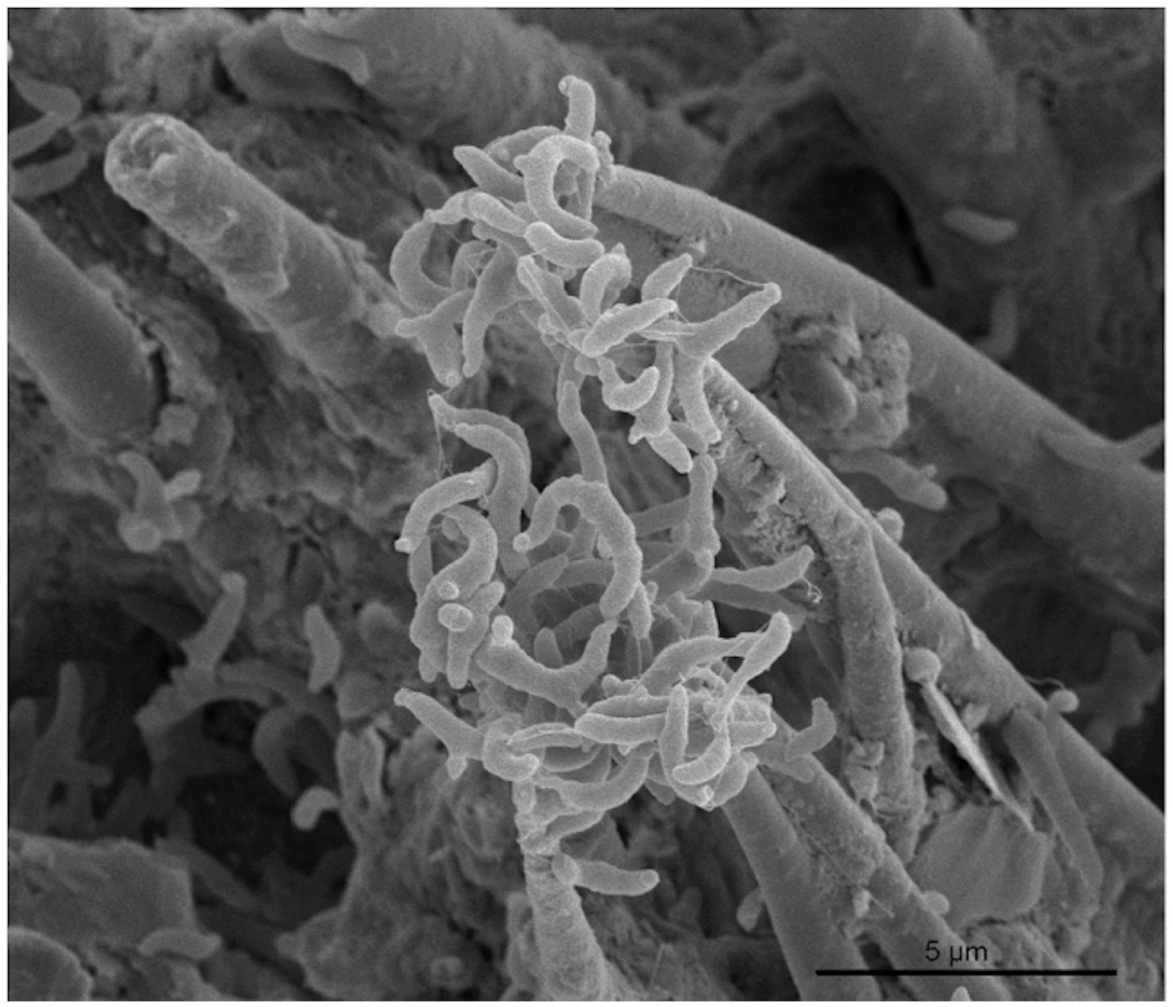
SEM image of a *Vibrio cholerae* biofilm on chitin from crab shells (Klose and Satchell, 2018). (Copyright Fitnat Yildiz, reproduced with permission).

Motility plays a key role in *Vibrio* biofilm formation. Specifically, motility is necessary for adherence and signal transduction that lead to proper development of the complex three-dimensional (3-D) biofilm structure (Yildiz and Visick, 2009). The *Vibrio* biofilm is a matrix-enclosed community capable of being formed on both abiotic and biotic surfaces, with preference for chitinous substrates (Figure 3) (e.g., zooplankton exoskeletons and marine snow) (Teschler et al., 2015). Bacterial biofilms protect the microbial community from antimicrobial substances, predation by bacteriophages and amoebae, and disperse nutrients to promote survival and environmental persistence (Donlan and Costerton, 2002; Yildiz and Visick, 2009). The complex 3-D structure of a mature biofilm is composed of microcolonies, forming pillars encased in dense exopolysaccharide, and fluid channels that disperse nutrients and diffuse out toxic substances (Costerton et al., 1994).

*Vibrio* biofilm gene expression is preferentially induced at low cell density, and regulation is partially controlled by quorum sensing (Yildiz and Visick, 2009). The concentration of the *V. cholerae* quorum sensing autoinducers CAI-1 and AI-2 (synthesized by CqsA and LuxS, respectively) present in the environment are recognized by the receptors CqsS and LuxPQ. The quorum sensing pathway is also regulated by the newly discovered receptors CqsR and VpsS, which currently have unidentified ligands (Jemielita et al., 2018). Cell density-responsive gene expression is regulated by the master regulators AphA and HapR. AphA, the low cell density (LCD) master regulator is active when LuxO is phosphorylated and regulatory RNAs (Qrr sRNAs) are produced. Qrr sRNA production also represses HapR, the high cell density (HCD) master regulator, which is otherwise active when LuxO is in its dephosporylated state. AphA regulates surface biofilms and virulence in *V. cholerae* (Jemielita et al., 2018).

Regulation of the *Vibrio* biofilm is also controlled by two-component signal transduction (VpsR, VpsT, and VarS-VarA), alternative sigma factors (RpoS, RpoN and RpoE) and c-di-GMP signaling (Yildiz and Visick, 2009; Teschler et al., 2015). The second messenger c-di-GMP is known for its ability to regulate the transition between a motile planktonic state to non-motile biofilm state for various bacteria (e.g., *S*. Typhimurium*, Caulobacter crescentus, Pseudomonas putida, and P. aeruginosa*) (Romling and Amikam, 2006). C-di-GMP inversely regulates motility and biofilm formation in *V. cholerae* (Liu et al., 2010; Teschler et al., 2015). Elevated c-di-GMP levels promote biofilm formation and inhibit flagellar mediated motility. Likewise, when c-di-GMP levels are decreased, flagellar mediated motility is promoted and biofilm formation is inhibited (Romling and Amikam, 2006; Yildiz and Visick, 2009). Several c-di-GMP modulating enzymes have been demonstrated to modulate *V. cholerae* motility, with the DGCs CdgD, CdgH, CdgK, and CdgL positively regulating motility, and the phosphodiesterase (PDE) CdgJ and DGC/PDE RocS negatively regulating motility (Liu et al., 2010).

Synthesis of the *Vibrio* biofilm requires structural components such as the flagellum, type IV pili (e.g., MSHA, TCP and ChiRP) and production of *Vibrio* exopolysaccharides (CPS/EPS/VPS) (Yildiz and Visick, 2009). In *V. cholerae*, the flagellum mediates near-surface roaming and orbiting modes of motility, which assist in the initiation of adhesion to a surface or to other bacteria (Utada et al., 2014; Teschler et al., 2015). Roaming motility is characterized by long, slightly curved trajectories over a large surface area. Orbiting motility is a mode of surface hovering, characterized as tightly-curved, circular trajectories over a small surface area. Roaming and orbiting modes of motility are inhibited in flagellar and MSHA pili mutants. Rotation of the polar flagellum allows MSHA pili to periodically contact a surface by counter-rotation along the cell body's major axis, referred to as surface skimming (Utada et al., 2014). Surface skimming also promotes microcolony formation as attachment can occur to the surface directly or to surface attached bacteria (Costerton et al., 1994).

In *V. cholerae*, the exopolysaccharide VPS (encoded by *vps* genes) is also regulated by the sodium-driven flagellar motor (Haugo and Watnick, 2002; Lauriano et al., 2004; Biswas et al., 2018). VPS expression is associated with the rugose colonial morphology phenotype, matrix production, formation of the 3-D structure of mature biofilms, pellicle formation and environmental persistence (Yildiz and Visick, 2009). The morphological switch from smooth to rugose can be observed in non-flagellated mutants of *V. cholerae*, indicating the loss of the flagellum induces VPS expression (Lauriano et al., 2004). Lack of the flagellum appears to be sensed via the flagellar motor because the introduction of a *mot* mutation into a non-flagellated *V. cholerae* mutant reduces VPS expression and converts back to a smooth colonial morphology (Lauriano et al., 2004; Biswas et al., 2018). This *Vibrio* motor-dependent VPS signaling pathway is reminiscent of the motor-dependent signaling pathway that controls lateral flagella expression in *V. parahaemolyticus* (McCarter, 2004). It has been postulated that flux of Na^+^ through the motor is required for the flagellar-dependent signal, since phenamil, a specific inhibitor of the Na^+^ motor, inhibits both *V. parahaemolyticus* lateral flagella and *V. cholerae vps* expression. Interestingly, many *V. cholerae* strains undergo the smooth to rugose switch when *hapR* is inactivated instead, and these strains do not appear to use the flagellum-dependent sensing mechanism (Lauriano et al., 2004). The HapR-dependent signaling pathway responsible for *vps* gene expression has been well-described (Beyhan and Yildiz, 2007).

*Vibrios* preferentially form biofilms on chitinous surfaces in the aquatic environment, and increased susceptibility to cholera infection has been attributed to consumption of *V. cholerae* bacteria encapsulated in the protective biofilm structure (Watnick and Kolter, 1999), which is more infectious than planktonic cells (Tamayo et al., 2010). Tamayo et al. (2010), suspect hyperinfectivity of *V. cholerae* biofilm cells is due to a physiological state the bacteria enter when associated in a biofilm, which is retained upon dispersal. *V. cholerae* dispersal from biofilms is aided by *rbmB*, a putative polysaccharide lyase, degradation of extracellular genomic DNA (eDNA) by the extracellular nucleases *dns* and *xds*, and the upregulation of motility genes (Teschler et al., 2015), again emphasizing the importance of motility in all aspects of the *Vibrio* lifecycle.

## Perspectives and Future Directions

*Vibrio* spp. are ubiquitous in the aquatic environment but are found in higher prevalence with increased water temperature. As global water temperatures continue to rise, there will be a consequential increase in the abundance of *Vibrios*. This will likely lead to an increase in *Vibrio-*associated diseases (e.g., coral bleaching, vibriosis in shellfish and mammals, and cholera infections). Because motility plays a critical role in the *Vibrio* lifecycle, a greater understanding of motility will yield insights into means to ameliorate the negative impact of *Vibrios* on humans and the environment.

A current gap in knowledge is the formation and importance of the flagellar sheath, one of the signature attributes of the *Vibrio* flagellum. The presence of LPS on the flagellum appears to be the basis for anti-LPS antibody-mediated protection against cholera (Bishop et al., 2010), and is critical for signaling during symbiotic interaction of *V. fischeri* with squid (Aschtgen et al., 2016), so understanding the basis of sheath formation will be important for the field. Likewise, the presence of multiple flagellin subunits within the filament is a signature of many polarly-flagellated bacteria, so elucidating the function of these additional flagellins will yield insight into motility of not just *Vibrios* but other polar flagellates as well. Many other novel aspects of the structure-function of the *Vibrio* flagellum are likely to be revealed by further study.

Understanding the manner in which motility and chemotaxis contribute to and are integrated into the virulence of various *Vibrio* species may lead to novel therapeutics and preventives. For example, determining how *V. coralliilyticus* uses chemotaxis to infect coral may lead to novel means to inhibit coral bleaching. The repression of flagellar synthesis during virulence and biofilm formation of some *Vibrio* spp. suggest that expression of motility is deleterious to these processes, so uncovering the basis of the inverse relationship between motility and virulence in *V. cholerae* and other Vibrios may unveil mechanisms by which bacteria can be induced to not cause disease by enhancing motility. A number of non-motile bacterial pathogens are capable of causing disease as well, so comparative studies of the virulence strategies utilized by flagellated Vibrios vs. non-flagellated pathogens would likewise be illuminating. Undoubtedly many incredible discoveries regarding the flagellum and its role in the *Vibrio* lifestyle will be revealed by the talented scientists studying this fascinating group of bacteria.

## Author Contributions

All authors listed have made a substantial, direct and intellectual contribution to the work, and approved it for publication.

### Conflict of Interest Statement

The authors declare that the research was conducted in the absence of any commercial or financial relationships that could be construed as a potential conflict of interest.
